# Cardioprotective effect of remote ischemic preconditioning with postconditioning on donor hearts in patients undergoing heart transplantation: a single-center, double-blind, randomized controlled trial

**DOI:** 10.1186/s12871-019-0720-z

**Published:** 2019-04-06

**Authors:** Guyan Wang, Ying Zhang, Lijing Yang, Yimeng Chen, Zhongrong Fang, Hui Zhou, Congya Zhang, Guiyu Lei, Sheng Shi, Jun Li

**Affiliations:** 1 Department of Anesthesiology, Beijing Tongren Hospital, Capital Medical University, Beijing 100730, China; 2 Department of Anesthesiology, Traditional Chinese Medicine Hospital of Southwest Medical University, Luzhou, 646000 Sichuan China; 3 Department of Anesthesiology, Fuwai Hospital, National Center for Cardiovascular Diseases, Chinese Academy of Medical Sciences and Peking Union Medical College, Beijing, 100037 China; 4 Department of Anesthesiology, Ruijin Hospital, Shanghai Jiaotong University School of Medicine, Shanghai, 200025 China

**Keywords:** Ischemia, Preconditioning, Postconditioning, Heart transplantation

## Abstract

**Background:**

The cardioprotective effect of remote ischemic preconditioning (RIPC) in cardiovascular surgery is controversial. This study investigated whether RIPC combined with remote ischemic postconditioning (RIPostC) reduces myocardial injury to donor hearts in patients undergoing heart transplantation.

**Methods:**

One hundred and twenty patients scheduled for orthotopic heart transplantation were enrolled and randomly assigned to an RIPC+RIPostC group (*n* = 60) or a control (n = 60) group. In the RIPC+RIPostC group, after anesthesia induction, four cycles of 5-min of ischemia and 5-min of reperfusion were applied to the right upper limb by a cuff inflated to 200 mmHg (RIPC) and 20 min after aortic declamping (RIPostC). Serum cardiac troponin I (cTnI) levels were determined preoperatively and at 3, 6, 12, and 24 h after aortic declamping. Postoperative clinical outcomes were recorded. The primary endpoint was a comparison of serum cTnI levels at 6 h after aortic declamping.

**Results:**

Compared with the preoperative baseline, in both groups, serum cTnI levels peaked at 6 h after aortic declamping. Compared with the control group, RIPC+RIPostC significantly reduced serum cTnI levels at 6 h after aortic declamping (38.87 ± 31.81 vs 69.30 ± 34.13 ng/ml, *P* = 0.02). There were no significant differences in in-hospital morbidity and mortality between the two groups.

**Conclusion:**

In patients undergoing orthotopic heart transplantation, RIPC combined with RIPostC reduced myocardial injury at 6 h after aortic declamping, while we found no evidence of this function provided by RIPC+RIPostC could improve clinical outcomes.

**Trial registration:**

Trial Registration Number: chictr.org.cn. no. ChiCTR-INR-16010234 (prospectively registered). The initial registration date was 9/1/2017.

## Background

The myocardium is susceptible to ischemia reperfusion injury (IRI) during cardiac surgery and known to be associated with adverse outcomes [1, 2]. The number of patients requiring heart transplantation will increase, and IRI is an inevitable consequence of heart transplantation [3, 4]. Although the long-term survival and quality of life of transplant recipients have improved significantly, strategies for improving myocardial protection and perioperative mortality rates have not substantially changed [5]. Therefore, efforts continue to devise an effective myocardial protective strategy for patients undergoing heart transplantation.

In early clinical studies, compared with ischemic preconditioning, remote ischemic preconditioning (RIPC) can be implemented through a simple, inexpensive and non-invasive technique, such as using a pneumatic cuff to cause transient limb ischemia [6]. In previous clinical trials, RIPC exerted a powerful protective effect on myocardial injury and significantly attenuated postoperative troponin increases in congenital cardiac, abdominal aortic, cardiac valve and coronary artery bypass graft (CABG) surgery [6–8]. In addition, RIPC and remote ischemic postconditioning (RIPostC) protocols are likely to provide additive protective effects. In this regard, a previous study showed that in patients undergoing off-pump CABG surgery, compared with the control group, RIPC combined with RIPostC reduced postoperative serum cTnI elevations, whereas RIPC alone did not markedly alter the results [9]. In a study of experimental animals, Andreka et al. [10] found that RIPostC also provided a cardioprotective effect. In clinical practice, Hong et al. [11] concluded that the protective effect exerted by combined preconditioning and postconditioning reduced serum cTnI elevations in patients receiving off-pump CABG surgery by 48.7%. Although RIPC+RIPostC is expected to exert an effective protective effect, no previous study has investigated the effect of RIPC +RIPostC on heart transplantation.

We therefore hypothesized that RIPC with RIPostC would reduce myocardial injury in donor hearts and improve clinical outcomes in patients undergoing heart transplantation.

## Methods

This was a single-center, prospective, double-blind, randomized controlled trial. This study was registered in the Chinese Clinical Trial Registry (http://www.chictr.org.cn) with registration number ChiCTR-INR-16010234. The study was conducted in accordance with the principles of the Declaration of Helsinki and approved by the Medical Ethics Committee of Fuwai Hospital. Written informed consents were obtained from all participants prior to enrollment.

Patients aged 18 to 70 years old who had end-stage heart disease and were scheduled for primary orthotopic heart transplantation between January 2017 and August 2018 at Fuwai Hospital, Beijing, China were screened and considered for random allocation. The exclusion criteria were preoperative mechanical circulatory support, peripheral vascular disease affecting the upper limbs, and redo heart transplantation. Moreover, patients taking antidiabetic sulphonylurea or glibenclamide were also excluded because these agents have been shown to abolish the effects of ischemic preconditioning [12].

On the day of surgery, eligible patients were randomly allocated to receive either RIPC+RIPostC or sham RIPC+RIPostC (control) before heart transplantation. Randomization was performed using opaque envelopes that concealed the group allocation. A research fellow who was not involved in medical treatment or data analysis performed the enrollment, group assignment, and intervention. Patients, cardiac surgeons, and postoperative intensive care staff were all blinded to treatment allocation.

Remote ischemic conditioning was applied after anesthesia induction (RIPC) and 20 min after aortic declamping (RIPostC) and consisted of four 5-min cycles of right upper limb ischemia induced by a cuff inflated to 200 mmHg with an intervening 5 min of reperfusion during which the cuff was deflated. Patients in the control group underwent sham placement of the cuff around the right upper arm without inflation. Blood samples were collected to measure serum cardiac troponin I (cTnI) levels before surgery and at 3, 6, 12, and 24 h after removal of the aortic cross clamp.

Premedication, anesthesia, perfusion, cardioplegia, and surgical techniques were standardised. Electrocardiography (ECG), pulse oximetry, nasopharyngeal and bladder temperature, arterial blood pressure, central venous pressure, and pulmonary artery pressure were continuously monitored. Anesthesia was induced with intravenous etomidate (0.2–0.3 mg/kg), cisatracurium (0.2–0.3 mg/kg) or rocuronium (0.6–1.5 mg/kg), sufentanyl (1–2 μg/kg), and midazolam (0.2–1 mg/kg) and maintained with propofol (0.05–0.08 mg/kg/min), sufentanyl (300 μg–500 μg) and muscle relaxants (10 mg/h). A low concentration of sevoflurane (0.5–1%) was used if necessary (during central line implantation).

Orthotopic heart transplantation was conducted through median sternotomy. Standard non-pulsatile cardiopulmonary bypass (CPB) with a membrane oxygenator was used. During CPB, moderate systemic hypothermia (nasopharyngeal temperature 28 °C) was maintained. The recipient’s heart was removed. Orthotopic heart transplantation was performed using a double-venous technique. All patients received basiliximab (20 mg) before incision for immune induction. Methylprednisolone (500 mg) was administered (250 mg before incision and 250 mg after aortic declamping).

The primary endpoint was to compare serum cTnI levels at 6 h after aortic declamping between the two groups. Secondary endpoints were comparisons of serum cTnI levels at 3, 12, and 24 h after aortic declamping and postoperative clinical outcomes, including in-hospital death, new onset stroke, renal failure requiring dialysis, mechanical circulatory support, arrhythmia requiring treatment, re-operation for any cause, gastrointestinal bleeding, mechanical ventilation time, ICU length of stay, and postoperative hospital length of stay.

The clinical outcomes were derived from the Society of Thoracic Surgeons (STS) database registry [13]. New onset stroke was defined as a new ischemic or hemorrhagic cerebrovascular accident with focal neurological deficit persisting > 24 h and confirmed by brain computed tomography imaging. Re-operation for any cause included re-exploration for bleeding and surgical reintervention. Mechanical circulatory support was defined as postoperative use of an intra-aortic balloon pump (IABP) or extracorporeal membrane oxygenation (ECMO). Inotropic support was quantified by calculating the vasoactive inotropic score (VIS) from the mean dosage of inotropic drugs administered after CPB during surgery (Table 1) [14]. Arrhythmia requiring treatment included ventricular fibrillation, ventricular tachycardia, and atrial fibrillation requiring intervention. Data were obtained from medical records and reviewed by two cardiologists who did not participate in the study.

**Table 1:**
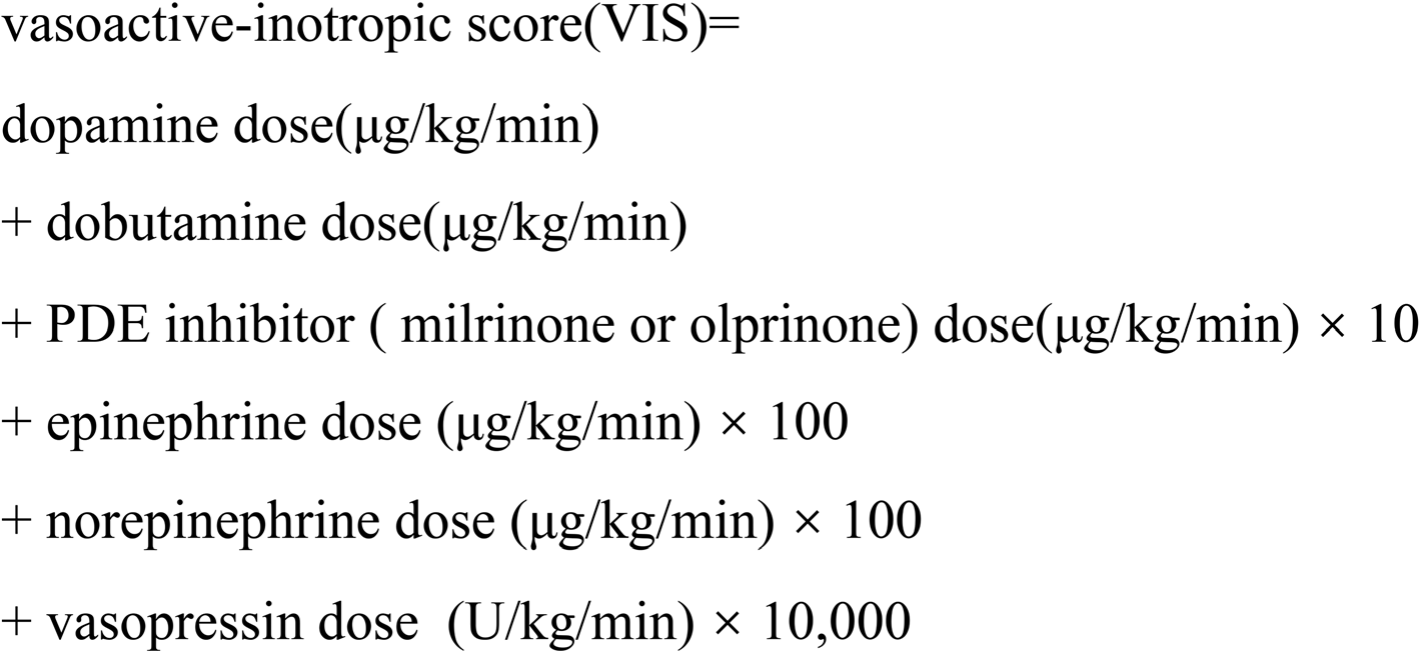
Formula for calculation of the vascoactive-inotropic score (VIS)

*VIS* vascoactive-inotropic score

### Statistical analysis of the data

The sample size was calculated according to our pilot study, and the serum cTnI level was 66 ± 23 ng/ml at 6 h after aortic decamping in the control group. We hypothesized that RIPC with RIPostC would significantly reduce serum cTnI levels by 30% and assumed a 5% dropout rate. To achieve 80% power at a two-sided significance level of 5%, a total of 120 patients was needed.

All statistical analyses were performed using SPSS version 20.0 (IBM Corp, Armonk, NY). The Shapiro-Wilk test was used to assess the normality of the distribution. For normally distributed data, all data were described as the mean ± SD. Nonparametric data were described as the median and interquartile ranges, and categorical data were described as the number of patients and the relative frequency per patient. Normally distributed variables were compared between groups with an independent-sample T test. Continuous variables that were not normally distributed were analysed with a nonparametric test (Mann-Whitney *U*). Categorical variables were compared between groups with the Chi-square test or Fisher’s exact test if the resulting matrixes contained cells with an expected count < 5. All tests were two-sided, and a *p* value of *P* < 0.05 was regarded as significant. This was an intention to treat analysis.

## Results

**Fig. 1:**
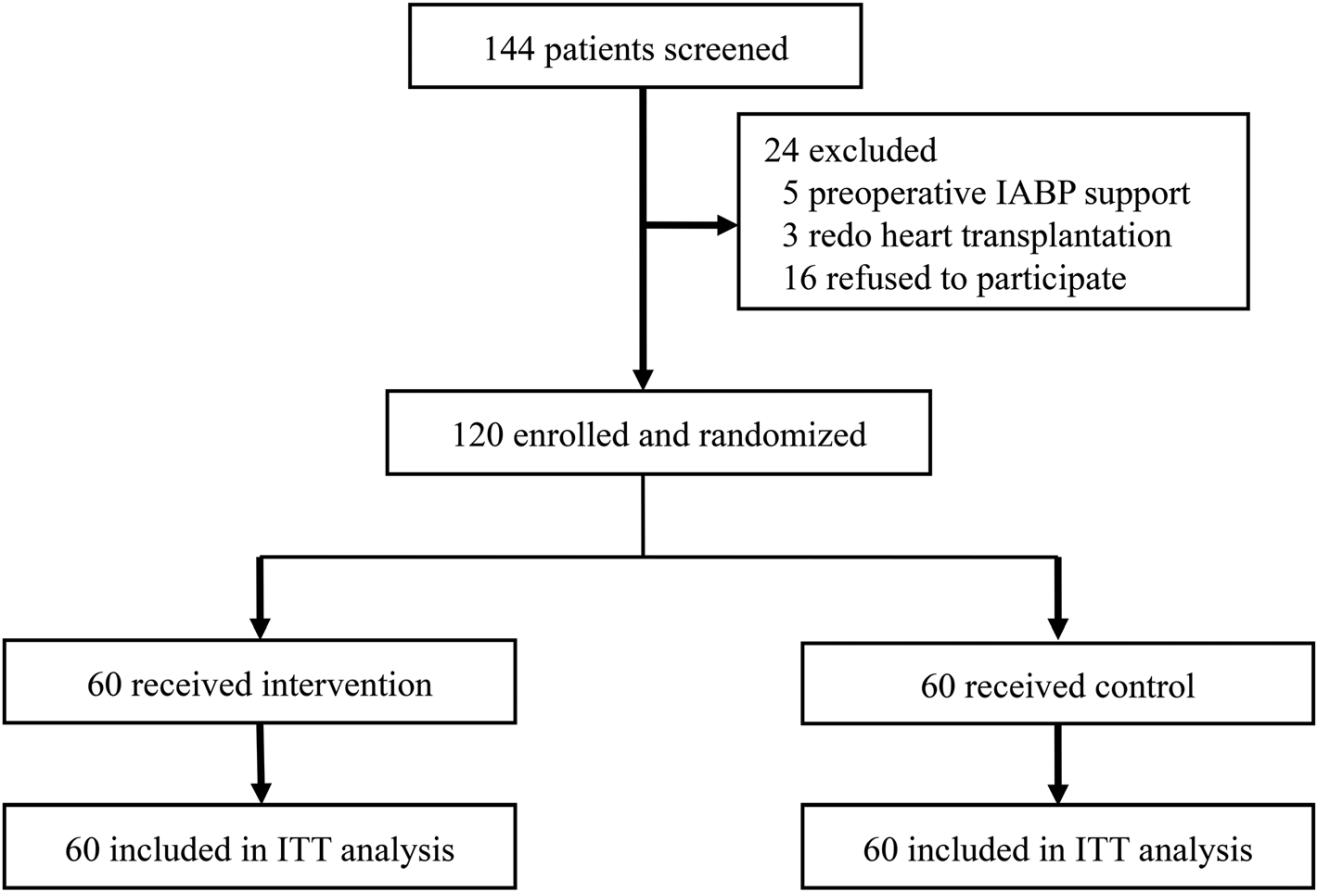
Flow chart. IABP, intra-aortic balloon pump; ITT, intention to treat

During the study period, 144 patients were screened for eligibility; of these, 120 met the inclusion criteria and were randomized to either the RIPC+RIPostC group (*n* = 60) or the control group (*n* = 60). Twenty-four patients were excluded (5 for preoperative IABP support, 3 for redo heart transplantation and 16 because they refused to participate) (Fig. 1).

The baseline characteristics of the two groups were presented in Table 2. There was no difference in donor heart ischemia time or the details associated with orthotopic heart transplantation surgery between the two groups (Table 3). No unintended effects or harm related to the RIPC+RIPostC procedure were detected.

**Table 2:** Baseline characteristics

	RIPC+RIPostC (*n* = 60)	Control (n = 60)	*P* value
Demographics			
Age (years)	46.5 ± 16.2	47.1 ± 12.4	0.983
Male	45 (75.0%)	44 (73.3%)	0.835
BMI	21.6 ± 3.7	21.4 ± 3.2	0.990
Distribution of primary diseases			
Coronary heart diseases	10(16.7%)	9(15%)	0.803
Cardiomyopathy			
Dilated cardiomyopathy	29(48.3%)	32(53.3%)	0.584
Hypertrophic cardiomyopathy	5(8.3%)	7(11.7%)	0.543
Restrictive cardiomyopathy	3(5%)	3(5%)	1
ARVC	6(10%)	3(5%)	0.488
Alcoholic cardiomyopathy	1(1.7%)	1(1.7%)	1
Peripartum cardiomyopathy	0	1(1.7%)	1
Noncompaction of ventricular myocardium	2(3.3%)	1(1.7%)	1
Valvular heart disease	3(5.0%)	2(3.3%)	1
Behcet disease	1(1.7%)	0	1
Myocarditis	0	1(1.7%)	1
Risk factors and comorbidities			
Hypertension	7 (11.7%)	6 (10%)	0.518
Diabetes mellitus	13 (21.7%)	10 (16.7%)	0.487
Hypercholesterolemia	12 (20%)	12 (20%)	1
Previous myocardial infarction	6 (10%)	4 (6.7%)	0.509
Previous stroke	6 (10%)	6 (10%)	1
Previous atrial fibrillation	20 (33.3%)	20 (33.3%)	1
Previous cardiac surgery	6 (10%)	8 (13.3%)	0.570
Cardiac status			
Left-ventricular ejection fraction (%)			
> 55%	4(6.7%)	3(5%)	1
35–55%	8(13.3%)	12(20%)	0.327
< 35%	47(78.3%)	45(75%)	0.666
Previous pacemaker	18(30%)	16(26.7%)	0.685
Preoperative medication			
Warfarin	8(13.3%)	12(20%)	0.327
β blocker	50(83.3%)	52(86.7%)	0.609
Lipid-lowering agent	3(5%)	8(13.3%)	0.114
ACE inhibitors or ARB	30(50%)	11(18.3%)	< 0.001
Aldosterone receptor blocker	46(76.7%)	57(95%)	0.803
Digitalis	16(26.7%)	49(81.7%)	< 0.001
Nitrates	5(8.3%)	12(20%)	0.670
Anti-diabetic drugs	6(10%)	8(13.3%)	0.570
Inotropic drugs	23(38.3%)	46(76.7%)	< 0.001

Data are mean ± SD or number (%)

*BMI* body mass index, *ARVC* arrythmogenic right ventricular cardiomyopathy, *ACE* angiotensin converting enzyme, *ARB* angiotensin-II-receptor blocker, *NYHA* New York Heart Association, *RIPC* remote ischemic preconditioning, *RIPostC* remote ischemic postconditioning

**Table 3:** Intraoperative characteristics

	RIPC+RIPostC (*n* = 60)	Control (*n* = 60)	*P* value
Length of surgery (h)	5.9 ± 1.2	6.1 ± 1.4	0.40
Donor heart ischemia time (h)	5.3 ± 1.9	4.9 ± 1.6	0.19
CPB time (min)	215.3 ± 49.5	233.2 ± 64.4	0.09
Aortic cross-clamp duration (min)	70.7 ± 18.1	74.8 ± 17.5	0.21
Reperfusion time (min)	130.3 ± 34.0	139.3 ± 43.7	0.21
Defibrillation after aortic declamping	18 (30%)	12 (20%)	0.23
Intraoperative ECMO	3 (5%)	3 (5%)	1
The use of sevoflurane	3(5%)	4(6.7%)	1
VIS after CPB	8.8 ± 6.1	9.3 ± 6.1	0.60

Data are mean ± SD or number (%)

*CPB* cardiopulmonary bypass, *ECMO* extracorporeal membrane oxygenation, *RIPC* remote ischemic preconditioning, *RIPostC* remote ischemic postconditioning;

*VIS* vascoactive-inotropic score

### Myocardial injury

Baseline preoperative serum cTnI levels were comparable between the two groups. Serum cTnI levels significantly increased in both groups after the operation procedure and peaked at 6 h after aortic declamping. RIPC+RIPostC significantly reduced peak cTnI levels (at 6 h) (38.87 ± 31.81 vs 69.30 ± 34.13 ng/ml, *P* = 0.02). There was no significant difference in the serum level of cTnI at the other postoperative time points (Table 4).

**Table 4:** cTnI levels

	RIPC + RIPostC (*n* = 60)	Control (n = 60)	*P* value
T1 (before surgery)	0.05(0.26, 0.11)	0.04(0.02, 0.97)	0.34
T2 (3 h after aortic declamping)	44.08 ± 32.19	51.99 ± 36.53	0.26
T3 (6 h after aortic declamping)	38.87 ± 31.81	69.30 ± 34.13	0.02
T4 (12 h after aortic declamping)	33.64 ± 31.79	43.7 ± 32.95	0.13
T5 (24 h after aortic declamping)	30.17 ± 26.34	31.40 ± 26.21	0.74

Data are number of median(quartiles) or mean ± SD. cTnI, cardiac troponin I; RIPC, remote ischemic preconditioning; RIPostC, remote ischemic postconditioning

### Clinical outcomes

There were no significant differences in in-hospital mortality, length of ICU stay, mechanical ventilation time, or other clinical outcomes between the two groups (Table 5).

**Table 5:** Postoperative characteristics and clinical outcomes

Variables	RIPC+RIPostC (*n* = 60)	Control (*n* = 60)	*P* value
Length of ICU stay (d)	3.9(3, 5.8)	4(3, 6)	0.63
ICU stay >7d	10(16.7%)	8(13.3%)	0.61
ICU stay >14d	6(10%)	4(6.7%)	0.51
Mechanical ventilation time (h)	35(22, 44.5)	28.5(22, 41.8)	0.39
Mechanical ventilation time > 48 h	12(20%)	11(18.3%)	0.75
Mechanical ventilation time > 72 h	9(15%)	4(6.7%)	0.13
Postoperative hospital stay (d)	16(13, 22.5)	15(12.3, 22.8)	0.81
Postoperative hospital stay >28d	11(18.3%)	6(10%)	0.19
In-hospital death	2(3.3%)	0	0.50
New onset stroke	1(1.7%)	0	1
Renal failure requiring dialysis	2(3.3%)	1(1.7%)	1
IABP support	4(6.7%)	6(10%)	0.51
ECMO support	3(5%)	2(3.3%)	1
Atrial fibrillation	3(5%)	0	0.24
Use of a temporary pacemaker	4(6.7%)	0	0.12
Arrhythmia requiring treatment	4(6.7%)	1(1.7%)	0.36
Re-operation	3(5%)	3(5%)	1
Re-intubation	1(1.7%)	1(1.7%)	1
Tracheotomy	1(1.7%)	1(1.7%)	1
Pulmonary infection	14(23.3%)	24(40%)	0.05
Deep sternal infection	3(5%)	0	0.24
Gastrointestinal bleeding	3(5%)	0	0.24

Data are median (quartiles) or number (%)

ICU, intensive care unit; IABP, intra-aortic balloon pump; ECMO, extracorporeal membrane oxygenation; RIPC, remote ischemic preconditioning; RIPostC, remote ischemic postconditioning

## Discussion

The present study is the first to demonstrate the effectiveness of RIPC combined with RIPostC in patients undergoing orthotopic heart transplantation. The results showed that compared to the control group, RIPC+RIPostC reduced serum cTnI levels in donor hearts at 6 h after aortic declamping, while we found no evidence that this effect of RIPC+RIPostC improved clinical outcomes after surgery.

RIPC prevents IRI and has myocardial protective effect, which was demonstrated by the very first RIPC study. Besides, as RIPC is simple, inexpensive and non-invasive, numerous studies have been done since its debut. In clinical studies, RIPC predominantly reduced postoperative myocardial enzyme levels in cardiovascular surgery patients. Hausenloy et al. [15] induced RIPC by inducing lower limb ischemia in patients undergoing on-pump CABG, and they found that this procedure attenuated myocardial injury at 6, 12, 24, and 48 h after surgery. While this effect was confirmed by our preceding meta-analysis (see Yang et al. [16]), in our study, mortality, morbidity, and other clinical outcomes were not improved. Additionally, several studies [17–19], including two multicenter randomized clinical trials with a larger sample size in cardiac surgery reported by Hausenloy et al. [18] and Meybohm et al. [19], failed to demonstrate that RIPC exerts beneficial effects in this type of patient.

There are several explanations for our results. This study tested the myocardial protective effect of RIPC+RIPostC in patients undergoing orthotopic heart transplantation, who may benefit highly from an effective preventive strategy. In the aforementioned studies (Hausenloy et al. and Meybohm et al.), most of the patients underwent CABG, and some patients underwent valve or ascending-aorta replacement or combined procedures. Postoperative complications are inherently less frequent in these types of surgery than in orthotopic heart transplantation. In addition, most patients who suffered from angina repeatedly have experienced the preconditioning of ischemia before CABG. Thus, the beneficial clinical effect of RIPC may be limited during CABG and valve or ascending-aorta replacement or combined procedures. Other clinical studies have shown that RIPC significantly reduced the rate of acute kidney injury (AKI), and renal replacement therapy was required in high-risk patients undergoing cardiac surgery [20]. It is highly likely that preconditioning is more effective in non-ischemic heart patients with a high risk and high complication rate than in patients without these factors.

There are several mechanisms by which RIPC and RIPostC techniques have been applied in orthotopic heart transplantation in animals. In a study of heart porcine transplantation, the researchers found that hind limb preconditioning in a recipient animal exerted a significant cardioprotective effect on the subsequently transplanted and denervated donor heart [21]. Furthermore, Konstantinov et al. [22] demonstrated that performing RIPC in the recipient animal via a K^+^ATP channel decreased IRI in the donor heart following orthotopic heart transplantation. RIPostC is thought to recruit a mechanism that is completely different from that of RIPC during the reperfusion period, and the mechanism induced by RIPostC remains to be determined [23]. Our results collectively suggest that humoural mechanisms play a crucial role when the combination RIPC with RIPostC technique is used in orthotopic heart transplantation.

Another possible confounding factor in our results was the use of propofol and sevoflurane for anesthetic maintenance. There was no significant difference in the use of sevoflurane in two groups in our study. Besides, the size of the sample, in which anesthesia maintained with sevoflurane, was very small (only 4 patients in the control group and 3 patients in the RIPC+ RIPostC group). Thus, the use of sevoflurane did not interfere with the results. The effect of RIPC and RIPostC may have been less affected by the use of propofol in our study than in other studies that used RIPC alone. Because the RIPostC protocol provides additive cardioprotective effects, it may offset a part of the negative effect of propofol. In addition, the dose of propofol was low (0.05–0.08 mg/kg/min) in the present study, and large doses of sufentanyl were administered. Overall, several promising effects were observed in this study even though propofol anesthesia was used. Recently, several basic studies [23] and clinical studies [24, 25] have reported conflicting results regarding the relationships among propofol, volatile anesthetics, and RIPC; thus, more evidence is needed to confirm that the influence of propofol and sevoflurane interferes with the protective effects of RIPC at different dosages and different operation types.

The number of patients using inotropic drugs preoperatively was significantly larger in the control group [46(76.7%) vs. 23(38.3%); *P* < 0.001]. One could argue that this could increase myocardial work, oxygen consumption, and potentially myocardial damage. All patients enrolled in this study were screened and randomly grouped*.* Furthermore, only low dose (3~5 μg/kg/min) dopamine or dobutamine infused by microinfusion pump was administered when patients required inotropic drugs before surgery in our center. Moreover, dopamine and dobutamine exhibited short half-life and rapid metabolism. Thus, the difference of the number of patients using inotropic drugs in two groups had little impact on the serum cTnI levels in patients 6 h after aortic declamping (the primary endpoint).

Our results showed that there was no significant difference in VIS (8.8 ± 6.1vs.9.3 ± 6.1; *P =* 0.60*)* between two groups after CPB. VIS is a score reflecting the amount of inotropic drugs support. If the intervention significantly affected myocardial protection, a lower inotrope requirement and a lower VIS after CPB would be expected. A possible explanation for this was the lack of appropriate samples, since VIS after CPB was not given at the primary endpoint. Furthermore, many factors affected the use of inotropic drugs during the operation, while the protective effect of RIPC combined with RIPostC could not significantly impact the VIS.

This study has some limitations. First, this was a single-center study that included a relatively small sample size of patients who participated in a randomized controlled trial. Orthotopic heart transplantation surgery is a highly complex procedure with high demands for both the surgeon and the anesthesiologist. It was therefore unrealistic to conduct a multicenter trial. Second, the serum cTnI level is a sensitive and specific biomarker for detecting cardomyocytes injury that can reflect the amount of myocardial destruction. Although we found that serum cTnI levels were significantly reduced in our study, the number of patients enrolled was too small and a larger-scale trial is needed. Third, long-term clinical outcomes were not investigated.

## Conclusion

The present study is the first to demonstrate the effects of RIPC with RIPostC in patients undergoing orthotopic heart transplantation. We found that applying RIPC with RIPostC reduced serum levels of the myocardial injury marker cTnI at 6 h after aortic decamping. Further studies performed to explore different clinical outcomes as primary endpoints and that include follow-up data are clearly warranted.

## Abbreviations

CABG: Coronary artery bypass grafting
cTnI: cardiac troponin I
IRI: Ischemia reperfusion injury
RIPC: Remote ischemic preconditioning
RIPostC: Remote ischemic postconditioning
